# Pennsylvania Hospital

**Published:** 1838-06-06

**Authors:** Henry H. Smith

**Affiliations:** Resident Surgeon


### CLINICAL REPORTS.

### PENNSYLVANIA HOSPITAL.

[*Reported by* Henry H. Smith, M. D., *resident Surgeon.*]

*Case of Amputation of the Arm, for a gun-shot wound near the elbow—Ligatures removed in eight days.*

Samuel N——, aged 16 years, whilst out on the 28th of March, with a companion, who was intoxicated, was shot in the arm by a marble, which had been placed in the gun instead of a bullet. The limb was dressed with an angular splint tightly bandaged, and the ball left in the wound. On the 29th, nearly twenty-four hours after the accident, he was brought to the Hospital. The arm was much swelled, inflamed, and painful; the ball had entered at the external condyle, and perforated the humerus at the thin part, above its articulating surface, and travelled to the back of the arm. The ball was extracted, a warm poultice placed around the joint, and the arm loosely bandaged in a carved angular splint. Ordered pulv. Dover. gr. x. at night, and f℥ss of mist. neut. every two hours.

*March 30th.* Slept tolerably; pulse quick; arm easier, and less inflamed; tongue dry and furred. Ordered salts and magnesia, low diet, and V. S. ad ℥x.

*March 31st.* Pulse one hundred in the minute; skin more moist; tongue less furred; less inflammation in arm; wound suppurating. Ordered calomel gr. iij. ipecac. gr. ss. every two hours.

*April 6th.* Arm has been suppurating kindly; inflammation much reduced; tongue clear and moist; pulse good. Simple cerate to wound.

*April 8th.* Attacked with erysipelas, which prevails generally in the ward; much swelling and inflammation around the wound. Ordered warm poultice to wound; arm left loose in splint; lead-water cloths to rest of arm, and free purgation.

*April 9th.* Wound sloughing; joint much inflamed, and swollen; tongue furred; considerable fever. Ordered same dressings, and blue mass gr. j., every two hours.

*April 10th.* Wound enlarged; free discharge of sanious matter; inflammation travelling upwards. Ordered poultice around the joint, sprinkled with pulv. canthar.; arm kept in splint; saline draught, and vegetable diet.

*April 11th.* Blister drew well; sloughing checked; wound three inches in diameter around the joint; discharging freely. Ordered simple poultice, with increased diet.

*April 13th.* Slough coming away; wound granulating. Continue treatment.

*April 16th.* Again attacked with erysipelas, for which the usual treatment was directed. This again produced sloughing, which removed the integuments around the joint, leaving an extensive ulcer. On the 23d he was attacked with diarrhœa and night sweats, and on the 25th of April, the inflammation having subsided, the arm was amputated by Dr. Randolph.

*May 2d.* The patient is improving in general health; wound looks healing, and is uniting as usual.

---

*Case of Dislocation of the Radius backwards.*

Mr. John B. W——, clerk, aged 23 years, whilst riding in the city, on the afternoon of the 12th of May, accidentally fell from his horse and dislocated the left radius backwards. He stated "that he fell on his left hand whilst it was turned inwards, and immediately felt a sharp pain in the elbow, which induced him to think he had broken his arm." He came at once to the Hospital; the arm was semiflexed; he was unable to supinate the hand, or to bend the elbow or straighten it, without intense pain. The head of the radius was distinctly felt behind the external condyle of the humerus, resting against the olecranon process. On partially pronating and supinating the hand, the head revolved distinctly, and owing to the little swelling, and the slight development of his muscles, the button shape of the radius was distinctly seen revolving against the olecranon. The patient, to use his own expression, had always been very "loose-jointed," but had never before dislocated any of his bones. After a close examination of the case, he was placed on a bed; an assistant was directed to seize the humerus near the condyles, place his thumbs against the head of the bone, and force it downwards and forwards. At the same time, I made extension at the wrist, suddenly straightened the arm, and supinated the hand, when the bone readily slipped into its place. The arm was then placed in a carved angular splint, loosely bandaged, and cold lotions applied to it. The next day, the patient could bend the arm and perform the usual motions of the hand, but, of course, was not allowed to do so. Little inflammation followed, and on the 14th of May, still wearing the splint, he returned to Mullaga Hill, N. J., where he resided.

This accident is one of very great rarity. Boyer says that he has seen but two cases; Duverney, three; and Sir Astley Cooper never met with it in the living, and but once in the dead subject. The closeness of the articulation, the strength of the muscles surrounding the joint, and dense fascia covering them, all tend to make it of rare occurrence. The light nature of the connection of the lower end of the bone to the bones of the wrist, by allowing it to glide over them, gives rise to frequent dislocations at this point and to fractures, by this means preventing the application of a direct force to the upper extremity of the radius, so that although we have in the course of a year, numerous dislocations of the lower extremity, yet this is the only one known for many years, of the upper portion of it. The state of the joint after the occurrence of the accident is minutely described by Boyer. Sir Astley Cooper, in the account of the case dissected at St. Thomas' Hospital, also furnishes an account of the state of the parts where the dislocation had been long unreduced. His directions for the reduction of the bone, would not, however, have availed in this case, for the arm might have been flexed to the utmost, without throwing the radius off the end of the humerus; but where the power is applied as recommended by Boyer, the reduction is immediate, as the force at the head of the radius throws it off the condyles of the humerus, whilst the extension of the arm, and the supination of the hand, must place the bone in its original position.

---

*List of Accidents, admitted into the Pennsylvania Hospital, from May 16th to May 30th,* 1838.

One case of fracture of the ribs, from a fall; dressed with a tight bandage. One case of rupture of external lateral ligament of the knee joint, caused by the sudden pressure of a bank of earth against a platform, by means of which the *leg* was forced inwards; the knee was leeched, the limb placed at rest in a fracture box, and cold applied constantly to it—doing well. One case of burn of the face and upper part of the chest, caused by blasting rocks; eyes slightly hurt; face kept moist by cloths wrung out of mucilage; collyria to eyes, and cerate to the chest. One case of fracture of the tibia and fibula, two inches above the ankle; dressed with fracture box.

The case of incised wound of the ankle, reported in the last, is cured. The tendo-Achillis united firmly in twenty-five days, and the patient has now the perfect use of his foot. The case of incised wound of the abdomen is well, and the man walking about.

